# A Novel Service-Oriented Professional Development Program for Research Assistants at an Academic Hospital: A Web-Based Survey

**DOI:** 10.2196/mededu.4576

**Published:** 2015-11-02

**Authors:** Robert Li Kitts, Kyle John Koleoglou, Jennifer Elysia Holland, Eliza Haapaniemi Hutchinson, Quincy Georgdie Nang, Clare Marie Mehta, Chau Minh Tran, Laurie Newman Fishman

**Affiliations:** ^1^Department of PsychiatryBoston Children's HospitalBoston, MAUnited States; ^2^Department of PsychiatryHarvard Medical SchoolBoston, MAUnited States; ^3^Division of ImmunologyBoston Children's HospitalBoston, MAUnited States; ^4^Department of Family MedicineSwedish First Hill ClinicSeattle, WAUnited States; ^5^Student AffairsMayo Medical SchoolRochester, MNUnited States; ^6^Department of PsychologyEmmanuel CollegeBoston, MAUnited States; ^7^Student AffairsTouro University Nevada College of Osteopathic MedicineHenderson, NVUnited States; ^8^Division of Gastroenterology and NutritionBoston Children's HospitalBoston, MAUnited States; ^9^Department of PediatricsHarvard Medical SchoolBoston, MAUnited States

**Keywords:** research assistant, research coordinator, community engagement, mentorship, preprofessional, training and development, workforce development

## Abstract

**Background:**

Research assistants (RAs) are hired at academic centers to staff the research and quality improvement projects that advance evidence-based medical practice. Considered a transient population, these young professionals may view their positions as stepping-stones along their path to graduate programs in medicine or public health.

**Objective:**

To address the needs of these future health professionals, a novel program—Program for Research Assistant Development and Achievement (PRADA)—was developed to facilitate the development of desirable professional skill sets (ie, leadership, teamwork, communication) through participation in peer-driven service and advocacy initiatives directed toward the hospital and surrounding communities. The authors hope that by reporting on the low-cost benefits of the program that other institutions might consider the utility of implementing such a program and recognize the importance of acknowledging the professional needs of the next generation of health care professionals.

**Methods:**

In 2011, an anonymous, Web-based satisfaction survey was distributed to the program membership through a pre-established email distribution list. The survey was used to evaluate demographics, level of participation and satisfaction with the various programming, career trajectory, and whether the program's goals were being met.

**Results:**

Upon the completion of the survey cycle, a 69.8% (125/179) response rate was achieved with the majority of respondents (94/119, 79.0%) reporting their 3-year goal to be in medical school (52/119, 43.7%) or nonmedical graduate school (42/119, 35.3%). Additionally, most respondents agreed or strongly agreed that PRADA had made them feel more a part of a research community (88/117, 75.2%), enhanced their job satisfaction (66/118, 55.9%), and provided career guidance (63/117, 53.8%). Overall, 85.6% of respondents (101/118) agreed or strongly agreed with recommending PRADA to other research assistants.

**Conclusions:**

High response rate and favorable outlook among respondents indicate that the program had been well received by the program's target population. The high percentage of respondents seeking short-term entry into graduate programs in health care-related fields supports the claim that many RAs may see their positions as stepping-stones and therefore could benefit from a professional development program such as the one described herein. Strong institutional support and sustainable growth and participation are other indications of early success. Further evaluation is necessary to assess the full impact of the program, particularly in areas such as job satisfaction, recruitment, retention, productivity, and career trajectory, but also in reproducibility in other institutions.

## Introduction

### Background

In 21^st^-century academic medicine, the prioritization of evidence-based clinical practice has led to the expansion of clinical and translational research and quality improvement projects. This shift has resulted in the increased hiring of an employee pool that includes research assistants (RAs), study coordinators, quality improvement assistants, and other similar positions (also identified in this paper as RAs) at academic hospitals [1]. Despite their essential role in the research team, RAs are often “invisible” when it comes to their own professional development [1-7]. A review of the medical literature demonstrates a lack of formal programming and career development structure directed toward RAs, which may lead them to feel isolated, undervalued, unrecognized, and undersupported in their work [1,4,7]. This lack of community, support, and recognition may compound additional job dissatisfaction related to the following: (1) low salary, (2) feeling unchallenged, and (3) not doing what they expected to be doing. Altogether, these factors may contribute to a decrease in motivation, performance, and retention [1,7,8], and may ultimately impact career trajectory.

Despite not being hired as trainees (eg, medical students, residents, and fellows), RAs may represent the next generation of medical and health care professionals [9]. The jobs they fill may serve as stepping-stones on their path to medical school and other graduate-level health care programs. As such, academic institutions that promote job satisfaction and professional development among this population may benefit not only from short-term research productivity, but also from long-term workforce sustainability. Interestingly, very little is written in the medical literature on such programs, suggesting that few, if any, exist [1,3,6,9].

The understanding that a large percentage of RAs at one particular academic hospital fit this demographic (ie, young adult, pregraduate) led to the development of such a program. This article details the impact that such a program can have on RAs' professional development as well as on the institutions they work for. By presenting the unique concept and the low-cost benefits of the program, the authors aim to encourage other hospitals to consider establishing similar programs.

### Establishment

In 2010, a team of RAs at a large pediatric teaching hospital, mentored by a faculty physician, established the Program for Research Assistant Development and Achievement (PRADA) for the purpose of creating a structured learning environment similar to the institution-wide programs already in place for trainees, postdoctoral and clinical fellows, and faculty. The steps taken to implement the program included the following: (1) developing a clear mission statement and identifying goals, (2) receiving institutional endorsement, (3) creating a product of value to recruit RAs, (4) empowering RAs to run and expand the program, and (5) forming a means to evaluate the program's efficacy and impact.

Once the mission statement and goals were outlined (see Textbox 1), the program sought institutional endorsement. Initial pushback exposed concerns about productivity and whether this was an attempt, on the part of the RAs, to form a union. Given that the programming was set to occur during the lunch hour and after normal work hours, and the program would not be a venue for voicing employment-related issues, perceived pressures were alleviated and endorsement was ultimately received.

**PRADA mission statement and goals.**

Mission statement

- To provide a structured and supportive learning community for research assistants, study coordinators, and other young professionals to develop into tomorrow's leaders in health care

Goals

- Enhance job satisfaction
- Create an inclusive environment
- Ease acclimation into an academic clinical environment
- Provide career insights through panel-style seminars
- Facilitate skills development through lectures, workshops, and formal presentation
- Foster a service-oriented mind-set and promote community engagement through service and advocacy endeavors directed toward the hospital and surrounding communities

The initial anchoring product and recruiting tool of PRADA was a monthly seminar series at which invited guest speakers would speak on topics of interest to RAs, including the following: (1) professional guidance and skills (eg, scientific writing, public speaking, stress management, and getting into graduate and professional school), (2) health care-related career talks given by panels of health care professionals (eg, physicians, medical students, nurse practitioners, clinical psychologists, and career researchers), and (3) relevant contemporary issues (eg, health care reform, disparities in health care, and interviewing high-risk patient populations).

Steadily increasing interest and participation prompted the creation of a unique email distribution list for PRADA. While used primarily as a way to broadcast programming, the email list would also serve as a tool for monitoring growth. Today, the list includes over 500 currently employed RAs at Boston Children's Hospital (BCH), over 50 RAs at neighboring institutions, and over 70 alumni (ie, former RAs) at medical schools and other health care professional training programs who expressed interest in continued involvement prior to leaving. Feedback, both solicited and unsolicited, from outgoing RAs indicated that many wished they had learned about the program earlier in their employment. This led to a partnership with the hospital's Department of Human Resources (HR), which now advertises the program throughout their hiring processes, not only to promote early engagement in PRADA, but also as a method to enhance recruitment.

### Organization

One of the earliest goals of the program was to have it run entirely by RAs. This was accomplished in 2012 when an executive board, overseen by two codirectors and their faculty advisor, was formed with representation from each of the following subcommittees: (1) Seminar Series and RA Grand Rounds, (2) Schoolwork Assistance (a novel, RA-run tutoring service for patients, and their siblings, who do not meet criteria for public school tutoring), (3) Social Volunteerism (social events with volunteering themes), (4) Pre-Med Track (events corresponding with the annual application timeline), (5) Public Health, Community, and Advocacy (engages RAs in activities related to health care legislation advocacy along with community health outreach and education with stakeholders within, and outside of, the hospital), (6) Membership and Communications, (7) Alumni Affairs (connects current RAs with alumni to provide them with insights into particular programs), (8) Science Writing Mentor Program (pairs RAs with student authors who need help revising and submitting manuscripts to the Journal of Emerging Investigators: an open-source, peer-reviewed journal that publishes original science research performed by middle and high school students), and (9) Interactive Music Program (RAs, in collaboration with Child Life Services and music therapists, provide interactive musical performances to patients).

### Sustainability

Despite the transient nature of RA employment, the structure of PRADA has remained stable and has seen continued growth and participation. To ensure a seamless transfer of responsibility across committee positions, new executive committee members are recruited from within subcommittees and new subcommittee members are recruited at events year-round. Low operational costs also contribute to PRADA’s sustainability. Today, the only expenses that PRADA incurs are from the lunches served at noon seminars and the cost of running an annual summer conference for youth interested in health care-related careers.

## Methods

In 2011, an anonymous quality improvement survey was administered to examine the demographics of PRADA’s target audience, level of participation in and satisfaction with the programming (data not provided), career trajectory of members, and whether PRADA was meeting its goals. Respondents were invited through the PRADA email distribution list, which contained 179 unique institutional email addresses at the time of the survey. Regarding career trajectory, participants were asked to answer the multiple-choice question, "In three years I would like to see myself in: same position, continued research with increased responsibility, graduate school, medical school, or other." Subjects who selected *other* were given the option to elaborate in a space below, accessible through branching logic. To determine if PRADA was meeting its goals, a list of specific questions were asked with the stated goals of PRADA in mind (see Table 1). Subjects were asked to select responses from a 5-point Likert scale with 1 representing *Strongly disagree*, 2 representing *Disagree*, 3 representing *Neither disagree nor agree*, 4 representing *Agree*, and 5 representing *Strongly agree*. All study data were collected and managed using Research Electronic Data Capture (REDCap) electronic data capture tools hosted at BCH [10].

## Results

A total of 69.8% (125/179) of invitees responded, with 100% (125/125) of them submitting completed surveys. A total of 5 out of 125 (4.0%) were excluded based on previously established exclusion criteria pertaining to employment status at BCH, leaving a final sample of 120. The majority of respondents were non-Hispanic white (89/114, 78.1%), were female (94/116, 81.0%), were 22-26 years old (101/115, 87.8%), had a bachelor’s degree-level education (105/119, 88.2%), and were hired within the 2 years of survey administration (99/117, 84.6%). Most respondents (94/119, 79.0%) were seeking higher education, reporting their 3-year goal to be in medical school (52/119, 43.7%) or nonmedical graduate school (42/119, 35.3%). Only 8.4% (10/119) aimed to be in a research position with increased responsibility, 5.9% (7/119) were unsure, and 4.2% (5/119) aimed for a change in job with no research involvement. An additional 2.5% (3/119) selected *other* and no respondents selected an option to remain in the same position.

Respondents agreed or strongly agreed that PRADA had made them feel more a part of a research community (88/117, 75.2%), enhanced their job satisfaction (66/118, 55.9%), provided career guidance (63/117, 53.8%), and connected them with research assistants in other fields (56/117, 47.9%). Additionally, 35.6% (42/118) agreed or strongly agreed that PRADA made them a better research assistant. Overall, 85.6% of respondents (101/118) agreed or strongly agreed with recommending PRADA to other research assistants. See Table 1 for selected outcomes of the quality improvement survey.

**Table 1:** Selected outcomes of the anonymous quality improvement survey administered to PRADA membership in 2011.

Survey item	Strongly disagree/ disagree, n (%)	Neither agree nor disagree, n (%)	Agree/strongly agree, n (%)
Would recommend PRADA to other research assistants (n=118)	4 (3.4)	13 (11.0)	101 (85.6)
Feel like part of research community (n=117)	11 (9.4)	18 (15.4)	88 (75.2)
Enhanced job satisfaction (n=118)	12 (10.2)	40 (33.9)	66 (55.9)
Career guidance (n=117)	16 (13.7)	38 (32.5)	63 (53.8)
Connect with research assistants in the other fields (n=117)	28 (23.9)	33 (28.2)	56 (47.9)
Connect with research assistants in the same field (n=118)	37 (31.4)	40 (33.9)	41 (34.7)
Become a better research assistant (n=118)	20 (16.9)	56 (47.5)	42 (35.6)
Connect with a mentor (n=116)	43 (37.1)	48 (41.4)	25 (21.6)

## Discussion

### Principal Findings

The PRADA survey team set out to collect data for the purpose of quality improvement, as well as to assess career trajectory, and determine whether it was meeting its goals. The high percentage of RAs (94/119, 79.0%) indicating a 3-year goal of being in graduate or medical school supports the original hypothesis that many RAs see their positions as temporary posts along their way to higher education and more advanced positions. The premise that these RAs may find such a program valuable was supported by the large percentage of RAs who participated (125/179, 69.8%) and the large percentage of those who agreed or strongly agreed with recommending PRADA to others (101/118, 85.6%). Additional outcomes demonstrated that PRADA was meeting its first goal—to improve job satisfaction—with 55.9% (66/118) selecting *agree* or *strongly agree* on the Likert scale for this item. There was some indication that PRADA is on its way to meeting its second and third goals—to create an inclusive environment and to ease acclimation into an academic medical setting—given that 75.2% (88/117) of respondents indicated that they felt more a part of a research community and 47.9% (56/117) indicated that PRADA helped them meet RAs in other fields. However, further investigation is necessary to determine whether or not this has changed over time. Determining whether the final three goals—to offer career guidance, to facilitate skills development, and to promote a service-oriented mind-set through community engagement and advocacy opportunities—are being met requires further investigation, but high ratings of related programming (data not included) indicates that PRADA is on the right track toward meeting them. In all, outcomes indicate moderate success for PRADA bearing in mind the survey was conducted in 2011, 1 year prior to the expansion of the program into what it is today.

Other observable indicators of success include a recent attempt to replicate the PRADA model at a neighboring Harvard-affiliated academic hospital by an alumnus, endorsement from the Boston Children’s Hospital Department of Human Resources, and inclusion of PRADA program materials in recruiting and hiring processes, which speaks to the value the institution sees in the program. In April 2015, the executive committee and the founding faculty physician received the 2015 Harold Amos Faculty Diversity Award, established to recognize and celebrate faculty who have made significant achievements in moving Harvard Medical School and Harvard School of Dental Medicine toward being a diverse and inclusive community.

### Limitations

Limitations include a small sample size taken primarily from one institution. Additionally, the survey, having been designed for quality improvement purposes, did not have a control group, nor did the survey team obtain a baseline satisfaction for comparison. Additionally, the survey could not compare its demographic distributions against the hospital's records to demonstrate inclusivity. However, recent collaborations with HR have made this possible going forward.

### Future Directions

In response to the limitations of the survey administered in 2011, PRADA's survey team created a more substantial, Institutional Review Board-exempt, longitudinal study of its membership to further evaluate whether its programming has a significant impact on job recruitment and retention, job satisfaction, commitment to the institution and/or the field of pediatrics, as well as career trajectory. Additional questioning is being employed to better assess PRADA's progress toward meeting its goals, and demographic data will be compared with HR records to ensure inclusivity. The team also plans to explore reproducibility at other academic institutions.

### Conclusions

This program model offers RAs many unique and diverse opportunities to contribute to initiatives that they are passionate about without interfering with their work responsibilities. Organizing and/or participating in such activities facilitates the acquisition of skill sets considered desirable by admissions committees and expected of trainees [11-12]. These include the leadership, administrative, and team-building skills necessary to run, sustain, evaluate, and improve a program. These skill sets also include working with under-represented populations, learning how to engage community stakeholders, taking part in public health education and advocacy, and providing mentorship to the youth that will one day replace them; this promotes a service-oriented mind-set that encourages them to give back to their communities and effect positive change in their everyday and professional lives. At the same time, members have the opportunity to interact with patients, present their research, and learn about the various career paths that lay ahead of them while forming lasting relationships with colleagues and mentors.

### Summary

PRADA is a low-cost, sustainable program that may improve overall job satisfaction by addressing areas of potential dissatisfaction for RAs. It also offers opportunities that promote growth, accomplishment, contribution, and corporate citizenship [8]. Preliminary data suggest that PRADA is moving in the right direction with its goals, but further evaluation is necessary to measure its full impact and the potential impact of such a program in other institutions. The authors encourage other institutions to consider implementing such a program as a means for improving the quality of training that RAs obtain on their paths toward becoming tomorrow's leaders in health care.

## Abbreviations

BCH: Boston Children's Hospital
HR: Department of Human Resources
PRADA: Program for Research Assistant Development and Achievement
RA: research assistant (also research coordinator, research technologist, quality improvement assistant, data coordinator, and other similar young professional positions within academic hospitals).
REDCap: Research Electronic Data Capture

## References

[1] Rickard CM, Roberts BL, Foote J, McGrail MR (2007). Job satisfaction and importance for intensive care unit research coordinators: Results from binational survey. J Clin Nurs.

[2] Cambron JA, Evans R (2003). Research assistants' perspective of clinical trials: Results of a focus group. J Manipulative Physiol Ther.

[3] Nishimura C, Takahashi R, Miyamoto S, Saito T, Kanemaru A, Liehr PR (2003). Lessons learned as a research assistant studying ambulatory blood pressure in elderly Japanese stroke patients. Nurs Health Sci.

[4] Roberts B, Eastwood GM, Raunow H, Howe B, Rickard CM (2011). The intensive care research coordinator position in Australia and New Zealand: Self-perception of professional development priorities and "best" and "worst" aspects of the position. A cross-sectional Web-based study. Intensive Crit Care Nurs.

[5] Hobson J, Jones G, Deane E (2005). The Research Assistant: Silenced partner in Australia's knowledge production?. J High Educ Policy Manag.

[6] Smith W, Salenius S, Cobb C, Marzan R, Sabina S, Beccaria L, Sitton-Petro T, Werner DL, Kamm M, Lenz S, Taylor T (2010). A survey of clinical research coordinators in the cooperative group setting of the American College of Radiology Imaging Network (ACRIN). Acad Radiol.

[7] Speicher LA, Fromell G, Avery S, Brassil D, Carlson L, Stevens E, Toms M (2012). The critical need for academic health centers to assess the training, support, and career development requirements of clinical research coordinators: Recommendations from the Clinical and Translational Science Award Research Coordinator Taskforce. Clin Transl Sci.

[8] Nohria N, Groysberg B, Lee L (2008). Employee motivation: A powerful new model. Harv Bus Rev.

[9] Kabakci I, Odabasi HF (2008). The organization of the faculty development programs for research assistants: The case of education faculties in Turkey. Turk Online J Educ Technol.

[10] Harris PA, Taylor R, Thielke R, Payne J, Gonzalez N, Conde JG (2009). Research electronic data capture (REDCap)--A metadata-driven methodology and workflow process for providing translational research informatics support. J Biomed Inform.

[11] Monroe A, Quinn E, Samuelson W, Dunleavy DM, Dowd KW (2013). An overview of the medical school admission process and use of applicant data in decision making: What has changed since the 1980s?. Acad Med.

[12] Koenig TW, Parrish SK, Terregino CA, Williams JP, Dunleavy DM, Volsch JM (2013). Core personal competencies important to entering students' success in medical school: What are they and how could they be assessed early in the admission process?. Acad Med.

